# Experience and lessons from health impact assessment for human rights impact assessment

**DOI:** 10.1186/s12914-015-0062-y

**Published:** 2015-09-16

**Authors:** Kendyl Salcito, Jürg Utzinger, Gary R. Krieger, Mark Wielga, Burton H. Singer, Mirko S. Winkler, Mitchell G. Weiss

**Affiliations:** Department of Epidemiology and Public Health, Swiss Tropical and Public Health Institute, P.O. Box, CH-4002, Basel, Switzerland; University of Basel, P.O. Box, CH-4003, Basel, Switzerland; NomoGaia, 1900 Wazee Street, Suite 303, Denver, CO 80202 USA; NewFields, LLC, Denver, CO 80202 USA; Emerging Pathogens Institute, University of Florida, Gainesville, FL 32610 USA

**Keywords:** Health impact assessment, Human rights impact assessment, Corporate development project, Industrial agriculture, United Republic of Tanzania

## Abstract

As globalisation has opened remote parts of the world to foreign investment, global leaders at the United Nations and beyond have called on multinational companies to foresee and mitigate negative impacts on the communities surrounding their overseas operations. This movement towards corporate impact assessment began with a push for environmental and social inquiries. It has been followed by demands for more detailed assessments, including health and human rights. In the policy world the two have been joined as a right-to-health impact assessment. In the corporate world, the right-to-health approach fulfils neither managers’ need to comprehensively understand impacts of a project, nor rightsholders’ need to know that the full suite of their human rights will be safe from violation. Despite the limitations of a right-to-health tool for companies, integration of health into human rights provides numerous potential benefits to companies and the communities they affect. Here, a detailed health analysis through the human rights lens is carried out, drawing on a case study from the United Republic of Tanzania. This paper examines the positive and negative health and human rights impacts of a corporate operation in a low-income setting, as viewed through the human rights lens, considering observations on the added value of the approach. It explores the relationship between health impact assessment (HIA) and human rights impact assessment (HRIA). First, it considers the ways in which HIA, as a study directly concerned with human welfare, is a more appropriate guide than environmental or social impact assessment for evaluating human rights impacts. Second, it considers the contributions HRIA can make to HIA, by viewing determinants of health not as direct *versus* indirect, but as interrelated.

## Correspondence

### Background

In recent years, governments, international institutions and civil society have pressed companies to show whether and how their actions might affect the human rights of populations surrounding their projects [1]. The process for identifying, preventing, mitigating and accounting for companies’ impact on human rights is now referred to as human rights due diligence [2]. Corporate actors have begun to attempt prognostic assessments of human rights impacts [3], but methodological guidance is needed.

Existing impact assessment frameworks do not provide human rights analysis. Environmental impact assessment (EIA), dating back to the 1970s, provides clear guidelines for predicting how human activity is likely to affect the natural environment [4]. However, EIA typically fails to link environmental impacts and distal social and health outcomes [5]. Social impact assessment (SIA) was, at its inception, limited by disciplinary exclusionism, and efforts to broaden its lens have lacked clear direction and structure [6, 7]. Current SIA guidance includes archaeological, touristic, infrastructural, institutional and psychological impacts with social effects [8]. In practice, only a handful of such inclusive SIA have been published, with most indicating an approach based on quantitative data of socioeconomic conditions of communities, augmented or pared down at the assessor’s discretion.

The more recent development of health impact assessment (HIA) [9, 10] recognises that human impacts, like environmental and social impacts, need to be understood within a circumscribed framework of analysis. HIA approaches fundamentally differ from EIA and SIA, as the latter two developed as permitting tools, while HIA served specifically to consider risks and remediable impacts. HIA integrates interdisciplinary interests with inclusive public health frameworks [11–13], and is concerned with short-term and long-term, as well as direct, indirect and cumulative interactions between an impacting policy, programme or project and human welfare outcomes. Moreover, HIA applies equity as one if its core values, emphasising the desire to reduce inequity that results from avoidable differences in the health determinants and/or health status between different population groups [14, 15]. As such, HIA represents a stepping stone towards human rights due diligence. This paper describes a human rights impact assessment (HRIA) conducted on an industrial agriculture project in a rural part of the United Republic of Tanzania, using HIA as a methodological guide.

### HIA as a stepping stone

The World Health Organization (WHO) defines HIA as “a combination of procedures, methods and tools that systematically judges the potential, and sometimes unintended, effects of a policy, programme or project on the health of a population, and the distribution of those effects within the population” [16]. HIA emerged from the 1980s concept of “healthy public policy,” through which health was seen as a product of both the physical environment and social behaviours [17]. Healthy public policy aimed to ensure that individuals and organisations had the information to choose between health-promoting and health-damaging policies. Hence, with roots in public health and policy-making, HIA embraces an inter- and multi-disciplinary approach that is designed to be cognisant of the ongoing changes that occur in societies and their living environments. The HIA applies qualitative and quantitative methods, drawing from social and natural sciences to examine a network of interactions that potentially result in positive and negative health outcomes in affected populations [1, 18–21]. The collection and analysis of health data from different sources, stakeholder involvement and field observations are typical methods applied for informing the evidence-base of an HIA [22–24]. The recognition that determinants of health, as well as mitigation measures, often fall outside the remit of the health sector, is another important feature of HIA, making it a tool for promoting intersectoral collaboration [25, 26]. Hence, HIA addresses some (but not all) right-to-health principles in consideration of a project before and after the occurrence of actual effects [27]. It is an iterative, non-linear and adaptable process [24, 26, 28]. Done well, HIA of large-footprint capital developments incorporates the direct and indirect effects of economic growth, in-migration, infrastructural developments and other factors affecting human health [26, 29, 30]. Attention to labour, environment, water, education, housing and discrimination acknowledge additional, non-health, issues that are intrinsically rights-related [31]. These are among the several content and design components of HIA that make it an appropriate precursor to HRIA.

### Where HRIA and HIA diverge

**Fig. 1:**
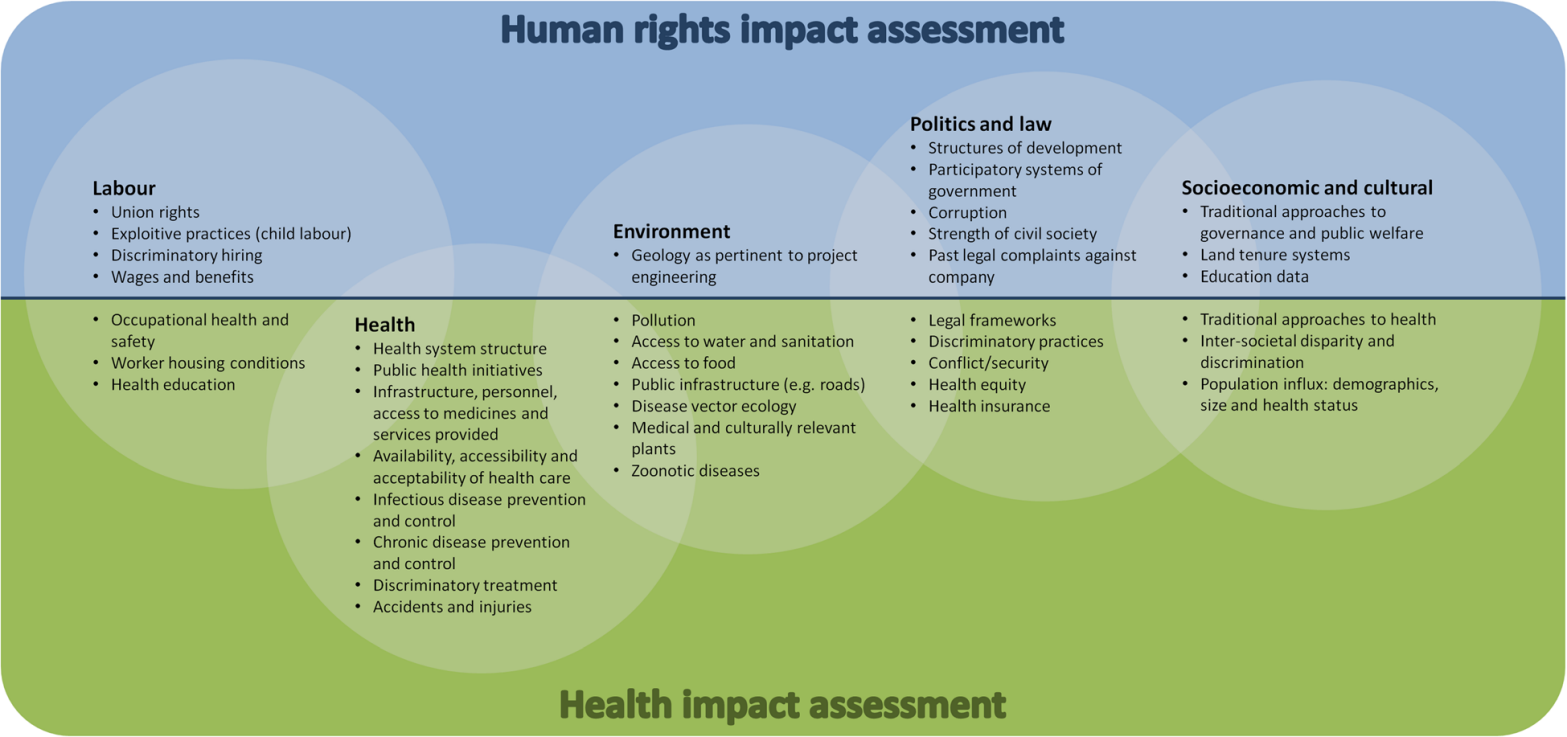
The range of interests and considerations pertinent to human rights impact assessment alone (*blue*) or both human rights impact assessment and health impact assessment (*green*). The authors have identified no interests and considerations within health impact assessment that are not also pertinent to human rights impact assessment

Yet, HIA does not expose all the human rights impacts of a project, programme or policy; that is not its aim. HIA regards causes of ill-health as proximal *versus* distal [32]. Though good-quality HIA should include the impact of policies and interventions on upstream causes of ill-health, health does not provide an inherent framework for recognising that the seemingly distal can directly affect health outcomes and secondarily affect the proximal [33]. HRIA does not start with a premise that certain determinants are likely to be secondary or tertiary, as all rights are considered equal and inseparable [34]. For instance, the natural environment might be a more relevant health determinant than the local economy when the local economy is subsistence farming and fishing, but local watersheds are polluted by upstream industry. In a potent example of the inappropriateness of the distal-proximal framework for right-to-health analysis, the 1973 US Supreme Court legalisation of abortion not only immediately improved access to reproductive rights for women and girls, despite being a federal court far removed from daily life, but it also had spinoff effects to legalise a range of service provisions in the vicinity of patients needing care [33]. Figure 1 depicts a contrast to Dahlgren and Whitehead’s rainbow diagram, featuring a rights-oriented organisation of topics relevant to welfare outcomes. This reorganisation maintains the importance of social structures, political frameworks, corporate social responsibility as well as community norms and personal characteristics – it views them as non-hierarchical, however. For example, national law might be a woman’s greatest barrier to reproductive rights in one country, while cultural norms might be the greatest barrier in another.

Additionally, HIA does not analyse how rightsholders will perceive the effects of a project or policy, an important aspect of human dignity, which is intrinsic to a human rights lens and imperative to understanding human welfare [35]. Local lore and beliefs are not often incorporated into HIA analysis. Conflicts over resource management, which are prevalent in project development, are fundamentally rights-related and crucially dependent on the perceptions of those involved [3, 36]. In contrast to HRIA, HIA does not involve soft-law compliance with international standards, which derives structure, and legitimises value judgments, from the instruments governing universal human rights.

Finally, there is an array of human rights that are impacted by project activities that do not pertain to health at all, but which are deeply relevant to human welfare and dignity. For example, the right to strike is not a topic analysed by HIA, but it is central to a rights-respectful workplace where employees have voice (and where it is violated, oppressive employers may use violence which can, indeed, affect health).

### Health under the banner of human rights

Health is not always considered in human rights terms, least of all during corporate health evaluations. Yet, health itself is specifically addressed in the International Bill of Human Rights, as a right “to the enjoyment of the highest attainable standard of physical and mental health”, benchmarked by standards of adequacy, affordability, availability, quality and cultural appropriateness [31]. Health is also defined by the principles of human rights, which include accountability, equity, participation and non-discrimination.

**Fig. 2:**
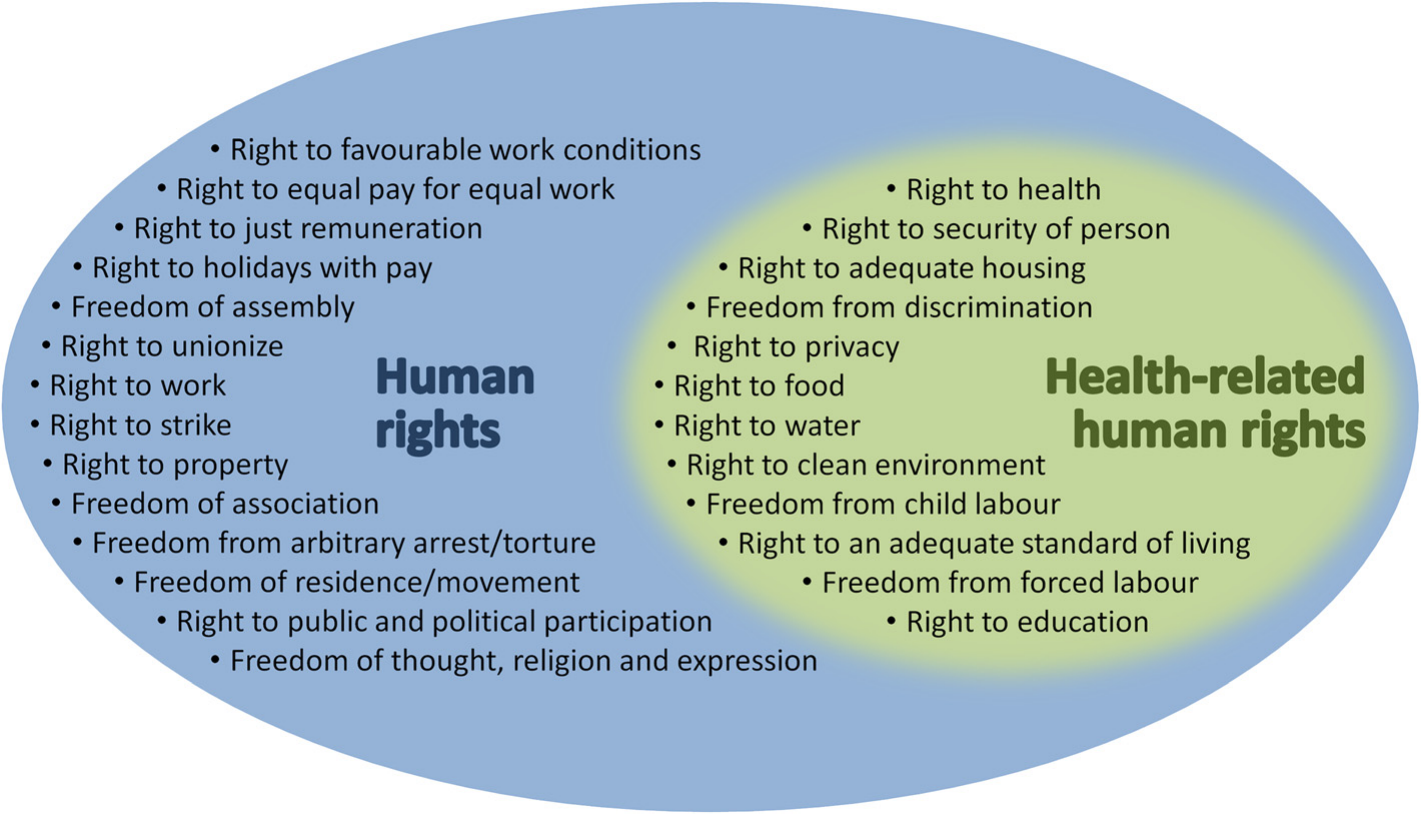
Health-related human rights as a subset of the full range of human rights

Right-to-health impact assessment, as distinct from HIA, is an important and growing field [37, 38]. Right-to-health impact assessment has made recent headway, investigated as a means for inequality and poverty reduction [38, 39], foreign policy [40], protecting public safety [41] and as a measurement of peace [42, 43]. Efforts to identify evidential links between human rights and health have been fruitful [44]. The task for human rights impact assessors is to use the broad understanding of challenging and complex systems that HIA uses on health networks to assess the entire suite of rights. The range of interests addressed in HIA, as they contribute to human rights evaluation, are depicted and grouped according to the topical organisation of HRIA in Fig. 2. This is not to say that HIA is subsumed, but rather that its expertise is incorporated into HRIA. In the same way, expertise of EIA, SIA and other project-commissioned studies contribute to HRIA. This allocation of thematic interests is represented in the topical groupings listed in Fig. 2.

## Methods: the HRIA approach

As health is the filter through which health impact assessors examine cultural, ecological, environmental, political and social conditions, so human rights is the filter for HRIA. Detailed descriptions of our HRIA methodology are available elsewhere [20, 45]. In brief, assessment entails scoping rights issues, cataloguing relevant topical inputs, scoring and rating impacts, issuing guidance and carrying out monitoring.

The process for vetting relevant content is standardised in impact assessment as scoping [24, 46]. Scoping, incorporating interviews, focus groups and document review, enables assessors to focus attention on certain human rights indicators, or topics, included in topic catalogues used for assessment. Scoping also identifies the need for supplementary analysis addressing specific situational concerns, triggering the use of particular modules. For example, in water-scarce contexts, a "water module" is incorporated, looking in greater depth at local water politics, allocation systems, quality, quantity, affordability and cultural dimensions than standard assessment would. Likewise, in countries with high HIV/AIDS rates or countries that have recently emerged from conflict, specific HIV and conflict modules can be used.

Scoping is followed by a cataloguing process whereby human rights relevant topics are considered, linked with relevant rights and rightsholders, and scored for the intensity and extent of impact. Roughly 300 assessment topics were developed using established indicators recognised in relevant fields, put forth by international organisations (*e.g.,* UNICEF and WHO [47]), standardised environmental monitoring indicators for environmental and social impact assessment (*e.g.,* NEPA), labour rights benchmarks through the International Labour Organization (ILO), and civil and political rights indicators developed by organisations such as Freedom House, Transparency International, the US Department of State and the Bertelsmann Transformation Index. These indicators primarily address contextual topics, while project- and company-related topics were developed to present likely changes from those baseline conditions. Project topics address the operation as designed and planned, including workforce needs, land and water usage estimates and interactions with government bodies. Company topics address the implementing corporation’s reputation, previous performance (in other contexts) and policy frameworks guiding operational decision making. These topic catalogues were refined through the piloting of HRIA on four continents in different industries, including petroleum, mining, energy, manufacturing and agriculture. Assessment topics are organised thematically, as depicted in Table 1.

**Table 1:** Human rights topics addressed during assessment, organised by broad subjects

Category	Sub-category	Rights topics
Labour	Wages	
	Unions	
	Exploitive practices	23 context topics
	Discrimination	20 project topics
	Labour laws	14 company topics
	Project employment profile	
Health	Health regulations	
	Underlying health determinants	
	Access and infrastructure	37 context topics
	Food	18 project topics
	Infectious diseases	9 company topics
	Risks to safety and health	
Environment	Surface water and groundwater	33 context topics
	Geology, ecosystem	21 project topics
	Air	5 company topics
Political and legal	Form of government	
	Strength of civil society	
	Law systems	34 context topics
	Strength of governance	18 project topics
	Non-discrimination regulations	10 company topics
	Civil war, conflict, security	
Economic, cultural and social	Demographics, local psychology	
	Economics	
	Indigenous peoples	32 context topics
	Education	29 project topics
	National culture	3 company topics
	Local cultures	
	Land the project occupies	

The column at right presents the number of topics analysed within each subject (adapted from: Salcito *et al.*, 2013 [21])

**Fig. 3:**
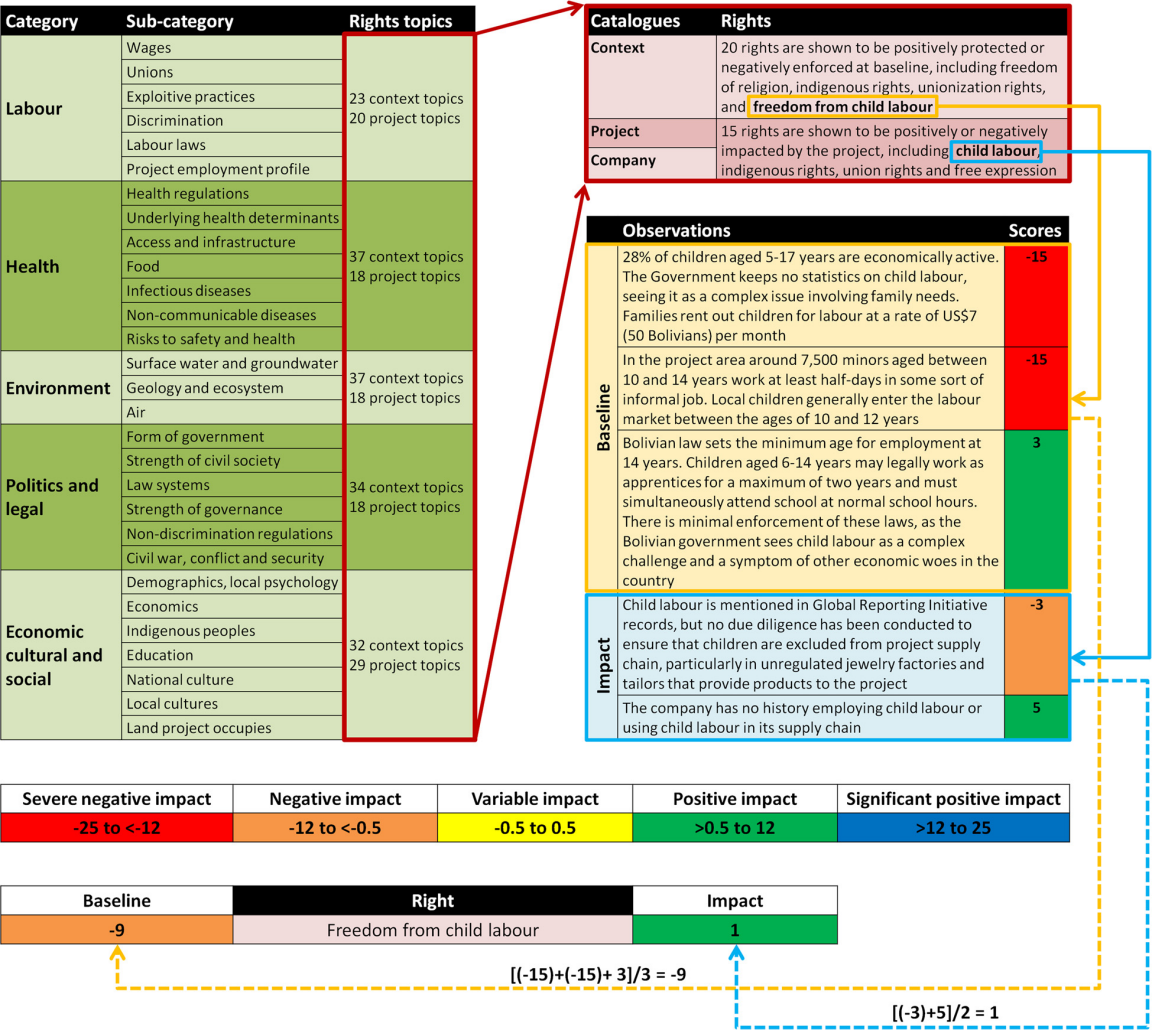
Flow chart of assessment and impact rating process, demonstrating the relationship between narrative descriptions of impacts and quantitative scoring

A scoring system weighing the intensity (severity for each affected rightsholder) and extent (number of rightsholders and degree of corporate complicity) of impacts establishes what topics to include in assessment. Extent of impact is not a designated number or percentage, but rather varies according to how many rightsholders exist and are affected within a certain subgroup of rightsholders. For example, if only two pregnant women are impacted by a policy, but there are only three pregnant women in the area, the impact has a high intensity on the particular rightsholder group. Likewise, if 100 working-age men are affected by an occupational harm, out of a workforce of 1000, the extent of impact remains considerable, even though it is not a majority (if it turns out that those 100 are a subgroup in themselves, perhaps an ethnic minority that is particularly susceptible to an effect, the impact retains a significant extent while also acquiring a higher intensity, as the severity of the effect on one subgroup is comparatively greater than on others). A right is assessed if intensity is greater than zero for its related topic. Actual assessment exposes the extent to which that impact is positive or negative [20]. These scores are sorted by human right and averaged to produce a rating ranging from −25 (extreme negative) to +25 (extreme positive). A flowchart of the process of scoring is depicted in Fig. 3. Finally, recommendations are issued and monitored in follow-up site visits.

## Experience and lessons from a case study in the United Republic of Tanzania

### Ethical clearance

Ethical clearance was sought from the ethics committee in Basel (EKBB), where the authors’ home institution is located (reference no. 304/13), as well as the National Institute for Medical Research (NIMR) (reference no. NIMR/HQ/R8a).

### Project selection

HRIA of the Green Resources Uchindile plantation in southern United Republic of Tanzania was undertaken to examine common and divergent interests of health impact analysis and the context of HRIA. Uchindile is located on the boundaries of Iringa and Kilombero districts, approximately 100 km from Iringa town, accessible on rough roads. It was selected for its rural location, where impacts could clearly be allocated to the project, not to third-party actors in the area, which did not exist when the project began in December 2008. It also has high poverty and infectious disease rates, low education and employment opportunities, and a growing migrant workforce. In short, the human rights baseline suffered from low state capacity to fulfil rights, and there were many ways in which the project could interact with existing human rights conditions, positively and negatively.

### Approach to evaluation

Uchindile plantation, founded in 2000, is owned and operated by Norway’s Green Resources AS. Assessors from NomoGaia, a non-profit think tank that builds and tests corporate human rights due diligence tools, examined likely impacts associated with the plantation’s transition from planting into harvesting. Initial assessment was timed to precede the transition to harvesting because a variety of workforce changes and health risks arise with the use of heavy machinery for tree felling and transport that are not needed during the growing and pruning stages of forest development. Assessment was continued periodically over the ensuing six years because changes in human rights conditions are ongoing. This is partly because one change in human rights conditions can trigger others (*e.g.* improved access to food can improve health outcomes), but also because harvesting operations occur over a shifting space – once trees are felled in one area, harvesters move to a different area. Furthermore, tree harvesters are semi-skilled workers, while the local area is populated by unskilled workers. Human rights impacts were considered possible as higher-paid workers were brought to the area to carry out paid work on land that was once held by local residents.

Green Resources provided interviews with all major management personnel (14 interviews over the course of three site visits) and a site tour. The assessment was not commissioned by the company and was externally funded by NomoGaia. The company was a willing collaborator in assessment, interested in human rights findings and willing to share data and facilitate interviews.

HRIA was carried out using the NomoGaia methodology, as described above, comprising scoping, cataloguing, scoring mitigation and monitoring [20]. Scoping entailed a systematic review of all publicly available audits, company financial reports, local and regional health and development reports and existing ethnographic studies in the Mufindi area. Certification reports, EIA, management plans, community questionnaires, annual reports and policy documents were studied as well as Tanzanian laws, Ministry of Health (MoH) reports and data from the national census and two Living Standards Measurement Surveys (LSMS) conducted in 2008 and 2010. A systematic search of all multinational publicly traded companies in Mufindi district, revealed foreign funding for the Mufindi paper mill and the presence of Unilever. Public documents pertaining to these sites were obtained to contribute to context analysis. Additionally, a Google Alert for “Mufindi,” “Iringa,” “Uchindile” and “Green Resources AS” between 2008 and 2014 alerted authors to news stories and activist reports during the assessment and monitoring period. Peer-reviewed literature in the fields of public health, economics, history and anthropology were drawn from a screening of authors’ personal collections as well as a Google Scholar screen for the same terms listed in Google Alerts. Additional national-level data were drawn from international databases, as standardised in the HRIA methodology (*e.g.* ILO, UNICEF, UN and WHO data). Data more than 10 years old and not from the Kilombero or Iringa districts were excluded. Data included reports from the grey literature to document both perceptions and misperceptions presented by outside observers and analysts without direct experience in the project area. All data were catalogued alongside sources, and all data were cross-checked during interviews with rightsholders, company personnel and local leaders, clinicians and other relevant authorities.

Cataloguing and monitoring involved primary data gathering and five site visits (March 2009, February 2010, November 2010, November 2013 and March 2014), each lasting between 5 and 10 days, involved engagement with health, education and government personnel (key informants) and rightsholders. Rightsholders are inhabitants of the project area whose human rights are likely to be impacted by project development and operations. Initial site visits represented a baseline from which observations in later visits were benchmarked. Rightsholder interviews were conducted with the most marginalised stakeholders, rather than with a random sample. Key informant interviews helped identify rightsholders experiencing disparate impacts. Semi-structured interviews asked informants to identify “outsiders,” people not considered part of the community and people not involved in community decision making. Particular probes were used to differentiate the power dynamics among men and women, first and second wives, locals and emigrants, and people of various educational attainment and skill levels. Interviews with women, youth, emigrants and other population subgroups enabled deeper exploration of relevant issues through a process of snowball sampling. Rightsholders included full-hire employees, contract labourers (both male and female), former employees, first and second wives of employees, the elderly, children, the ill, disaggregated for Kitete and Uchindile villages and plantation dormitories. Assessors also interviewed workers for job-specific impacts (*e.g.* fire guard, planters, pruners and nursery workers). Four feedback sessions with rightsholders, health personnel and project staff were held to verify findings. All interviews used semi-structured formats that allowed for digressions (sometimes extensive) onto topics deemed important by rightsholders.

Rights were scored through investigation of over 300 context-, project- and company-related topics, each linked to one of five thematic groupings associated with rights conditions, as shown in Table 1.

## Findings

### Human rights impacts

Initial assessment found positive impacts on the right to a clean environment and negative impacts on the right to water, working conditions, unionisation, remuneration, standard of living, housing, health, non-discrimination and education (Table 2). Rightsholders impacted included full-time employees, contract workers, women, the ill and children.

**Table 2:**
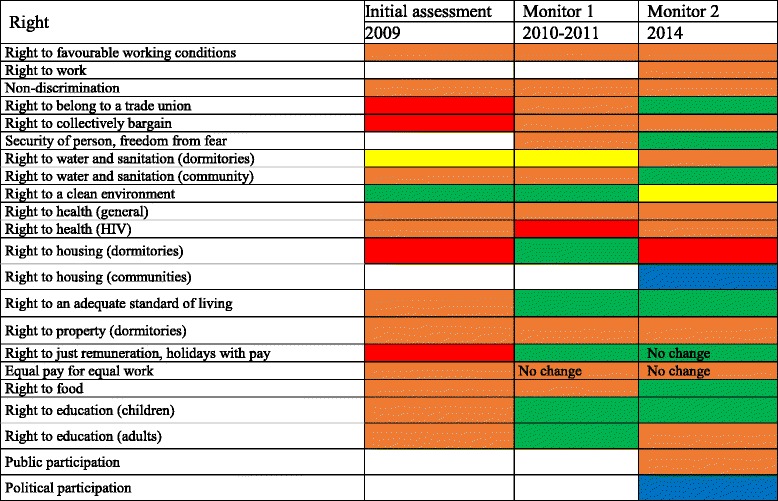
Human rights impact ratings at initial assessment in 2009, and follow-up monitoring in 2010/2011 and 2014

No change refers to cases where absolutely no conditions have changed. In some cases the colour ratings remain the same even if slight policy or procedural modifications resulted in numeric rating changes that did not affect colour scores (*e.g.* improvements that change an orange from a −8 rating to a −4 rating)

Human rights impacts overlapped with health impacts with regard to labour conditions, community welfare and project implementation. The company did not supply water to dormitories; instead, workers’ drinking water came directly from streams. Low wages inhibited workers’ ability to provide housing, clothing, healthcare and education to their families. Dormitories were rotting, had leaks and lacked space. At one housing bloc, 70 inhabitants were sharing 24 beds and two latrines. Workers reported being penalised for becoming pregnant and ill, including being assigned hard labour when health conditions would not permit such work. Maternity leave was available to 20 % of female workers. The project had no HIV policy or training programmes, which put it out of compliance with its government-approved development plan. The company’s failure to supply protective gear (*e.g.* for pesticide sprayers, who require respirators, goggles, gloves, boots and full-body coveralls) resulted in elevated injury rates above industry norms. Workers rode to fields on tractors, which, twice in one year, slid off muddy roads, injuring workers. Others walked 17 km to job sites. Project clinics suffered repeated stock outs of drugs and other medical equipment to treat work-related injuries. On two occasions assessors found clinics closed and unstaffed during site visits. No transportation was available to clinics, which were several kilometres away from worker housing.

Additional human rights impacts had no direct connection (although they had significant distal connection) to health. Wage equity appeared to be violated; women represented 20 % of the workforce but earned 17 % of total wages. Many workers could not file discrimination complaints, because, lacking literacy, they could not read grievance mechanism forms. Labour rights, including the right to unionise and collectively bargain, were restricted. For example, the union leader at Uchindile was removed from the plantation, leaving workers without a union liaison. Eighty per cent of the workforce believed they were ineligible for union participation, because, though most worked full time, they were hired as day labourers. Lacking job security, they did not feel empowered to demand better conditions or higher wages. Workers alleged that complaints resulted in dismissal.

In Table 2, red represents the most severe negatives, orange represents moderate negatives, yellow represents mixed impacts that have the potential to shift in either direction, green represents moderate positive impacts and blue represents significant positive impacts above and beyond the standard of “do no harm.” Boxes left blank represent impacts not registered at the time of assessment.

### Recommendations

Assessors cross-evaluated local conditions, industry standards (set by the World Bank and forestry initiatives) and human rights standards of adequacy (drawn from ILO, WHO and UN guidance). The following specific recommendations resulted:

- increase worker salaries to a living wage (approximately US$ 2/day);
- provide safety gear to all workers with penalties for non-usage;
- improve water access and quality using sand filtration;
- provide a minimum of three lorries to transport workers safely to project sites;
- increase number of beds, toilet facilities and dormitory capacity to accommodate all needed workers, and treat wooden construction materials to reduce rot and insect infiltration;
- develop and implement a comprehensive HIV/AIDS prevention and control programme;
- install solar panels at clinics to enable storage of antibiotics and provide light for emergency treatments needed after dark; and
- develop an anonymous, call-in grievance procedure to accommodate illiterate workers.

In content and form, these recommendations resemble basic public health interventions. The contribution of human rights was a governance framework that not only tied together the impacts so that the interacting effects of various working and living conditions could be better understood, but also that defined the company’s express responsibility to address each impact. This is important, because companies have a record of using corporate social responsibility initiatives as a way to address one public health problem, while they might be overlooking the deleterious effects they may be having at their own operations [48–50].

### First monitoring and mitigation

Initial assessment served to establish a baseline of corporate impacts, so that, going forward, company performance could be evaluated not only against human rights standards, but also against its previous positive and negative impacts. For example, an extreme negative impact on the right to health during initial assessment might be re-evaluated as a moderate negative impact on the right to health during monitoring. This would indicate that improvements had been made but the standard of adequacy set out in the UN guidance on the right to adequate, accessible, affordable, quality and culturally appropriate care had not been met. Such monitoring against previous performance is important, partly because many human rights impacts cannot be remedied immediately, and thus tracking activities that generate improvements are as important as tracking activities that generate rights-positive outcomes. Additionally, companies respond well to positive reinforcement and appreciate acknowledgement of the efforts they make as they internalise human rights.

Recommendations were issued directly to the company, to personnel at both headquarters in Norway and offices in the United Republic of Tanzania. Recommendations were published alongside full HRIA online at www.nomogaia.org, but they were not accompanied by advocacy activities. Despite the lack of advocacy activities, monitoring revealed a variety of improvements in human rights outcomes. Round one monitoring, conducted in November 2010 (20 months after initial assessment), documented several improvements in human rights conditions. Negatively scored impacts from initial assessment benchmarked improvement or deterioration in human rights conditions associated with each catalogued human rights topic. The company demonstrated positive impacts on the rights to adequate living standards, food, remuneration, housing and education. In several cases, workers who come from local villages (rather than live in dormitories) used supplementary income from recommended wage increases to upgrade houses. Project investment in a local school improved attendance and teacher retention rates. Insofar as classes were not interrupted by leaks and pupils were not at risk of injury within crumbling walls, conditions for learning improved.

Discriminatory conditions persisted. However, mitigation measures demonstrated progress. A manager who sexually harassed female workers was replaced. Work conditions remained difficult, and worker transportation problems had not been solved, but the company implemented midday meals, improved work conditions and the right to food. Equipment to protect workers against occupational hazards (*e.g.* protective boots, coveralls, gloves and masks for firefighting crews during dry season), became more widely available after assessment, reducing occupational health risks.

Management improved water access but continued to provide untreated water. Although several negative impacts on rights relevant to health were mitigated, ratings for the right to the highest attainable standard of health remained negative. The health rating associated with HIV dropped from negative to severe negative, as monitoring coincided with project relocation of workers from Iringa district (estimated HIV prevalence 15.7 % among men and women aged 15–49 years) [51] to Uchindile dormitories (estimated HIV prevalence 6 %) to conduct harvesting activities. The company has reported further improvements in human rights respect, which will be reviewed during a future site visit.

### Second monitoring and mitigation

A second monitoring evaluation, conducted 3 years later, with site visits in November 2013 and March 2014, evaluated whether mitigations had been sustained and/or new impacts had developed. Table 2 depicts that most changes from monitoring 1 were positive or neutral. Exceptions pertained to right to adequate housing for employees and favourable working conditions. Table 3 breaks down human rights impacts by rightsholder group, depicting that impacts became increasingly targeted to certain sub-populations.

**Table 3:**
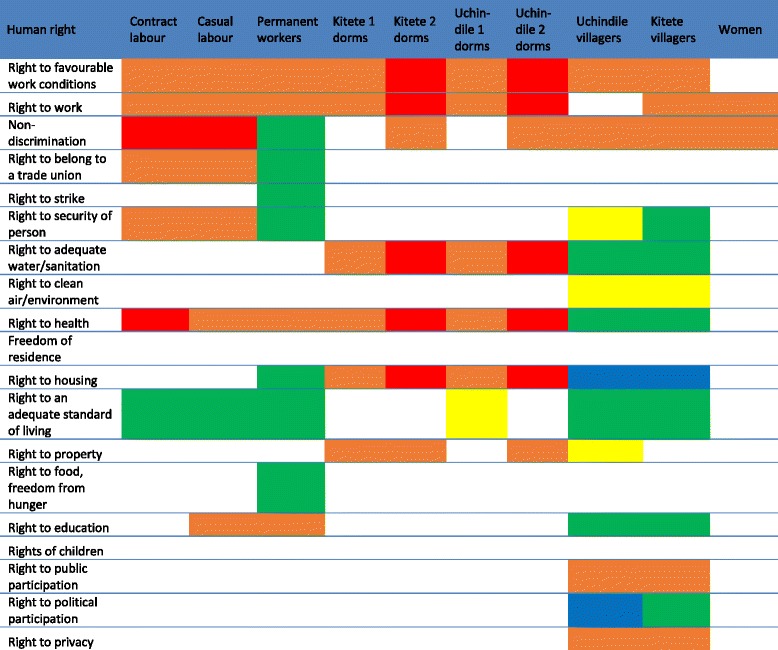
Human rights impacts disaggregated by rightsholder group, depicting the specific and divergent impacts projects have on diverse rightsholders

Blank boxes represent occasions where impacts were not registered for particular rightsholders

Major negative impacts surfaced for workers in Kitete and dormitories. Worker treatment had backslid, with decreasing access to transportation, a seasonal reduction to one daily meal, and considerable degradation of dormitories, including broken beds and disintegrating, unsanitary mattresses. The reversal suggested that human rights lessons had not been internalised, despite the company’s development of a human rights policy and reporting process.

However, major positive impacts were documented in Uchindile village, associated with living wage rates and political engagement. As the company distributed its first tranches of revenue from carbon sales, communities constructed and improved local infrastructure. Politicians came to appreciate the value of forestry in the region, triggering a debate over whether Uchindile should be redistricted into Iringa. In an effort to retain control of the area, Kilombero district authorities are increasingly attentive to the needs of Uchindile residents, improving boreholes, schools and clinics. For the first time in memory, Uchindile residents feel they have a voice at the district level. Additionally, continually increasing wages have enabled the majority of local area residents to improve private homes.

## Discussion

HRIA at the Uchindile plantation in the United Republic of Tanzania benefitted from HIA as a methodological guide. Contextual analysis of infectious disease prevalence and operations-level analysis of sanitation risks associated with poor water quality and insufficient latrines exposed multiple issues associated with the right to favourable working conditions and health. Without ensuring that the right to health was analysed using basic health indicators, intersecting rights issues would have been missed. This is notable because HRIA teams do not always include health workers. Corporations that have partnered with non-governmental organisations to assess their human rights impacts tend to focus on the legal and political risks, without recognising the interconnections among social determinants of health and spiralling human rights impacts that result from ill-health. Beyond the technical contributions of health analysis, there is a direct benefit that such assessments can have on human lives, by identifying risks to their welfare and targeting priority actions to mitigate or eliminate those risks. The study of human rights elucidated health issues which, in turn, revealed further human rights impacts associated with food, water, disease and occupational hazards, as well as non-discrimination, housing, living and labour standards [31, 52]. Increased mobility associated with harvesting activities was linked to potential impacts on the spread of HIV infection [53].

Monitoring revealed major improvements in several health-related human rights impacts, but the impact scores for the right to the highest attainable standard of health were unchanged. This suggests that facets of health may be more cohesively assessed under the umbrella of human rights than health. Several health impacts required non-health remedies, such as increased salaries, improved grievance mechanisms and management personnel changes. For example, workers replaced thatch roofs with corrugated iron sheets when salaries increased. Conversely, examination of education rights exposed health risks; the crumbling school the company promised to replace posed hazards to local children. Such right-to-health related risks were not immediately foreseeable through a health lens. Fully understanding right to health effects requires a broader human rights approach.

There is considerable overlap between health issues and human rights. HIA draws from environmental, health, labour and economic data to issue recommendations on health. HRIA draws from similar resources and frameworks, while broadening the investigation to incorporate civil, political, social and welfare rights. This process has the potential to enable companies to holistically address the risks and benefits they pose to the social-ecological systems where they operate.

A particularly noteworthy and under-discussed dimension of impact assessment is causality. While in this manuscript we have argued that the proximal and distal causes of health impacts are non-hierarchical, there is broad space for future research to consider the ways that the proximal and distal interact in a “chain of causation” or, more realistically, a web. In impact assessment, establishing the hierarchy of causes is often considered less vital than broadly identifying major causes. Hill puts forth nine tests for differentiating association and causation between environment and disease, before concluding ultimately that the idea is less to evaluate each test thoroughly than to ascertain enough information to best protect public welfare [54]. Impact assessment may not achieve a perfect chain of causation, but it should be sufficiently rigorous to lead assessors to recommend modified corporate actions [55].

## Conclusions

HIA is an increasingly accepted and established tool for identifying the impacts that corporate projects are likely to have on affected communities, while companies are increasingly being called upon to employ a broader “human rights lens” to their impact assessments. The same approaches that make HIA valuable – *i.e.* employing interdisciplinary research, generating concrete and actionable recommendations, basing findings on evidence – are needed in HRIA.

HRIA is increasingly expected of companies, builds on these techniques and augments them with perceptions and experiences of affected people. Our case study demonstrates the synergistic benefits of an intersectoral approach to impact assessment. The evidence-based approach of HIA, combined with consideration of “local knowledge” and experience, provides a framework for an HRIA that adds value to corporate assessments while meeting the expectations of the global community that they “do no harm.”

## Abbreviations

EIA: Environmental impact assessment
HIA: Health impact assessment
HIV: Human immunodeficiency virus
HRIA: Human rights impact assessment
ILO: International Labour Organization
LSMS: Living Standards Measurement Surveys
MoH: Ministry of Health
NIMR: National Institute of Medical Research
SIA: Social impact assessment
UN: United Nations
UNICEF: United Nations Children’s Fund
WHO: World Health Organization
